# Unethical research trend: shadow libraries

**DOI:** 10.1590/1516-3180.2017.0122210517

**Published:** 2017-08-21

**Authors:** Zeeshan Asim, Shahryar Sorooshian

**Affiliations:** I Doctoral Student, School of Industrial Management, Universiti Malaysia Pahang, Kuantan, Malaysia.; II Medical doctor and Senior Lecturer, School of Industrial Management, Universiti Malaysia Pahang, Kuantan, Malaysia

Dear Editor

Research articles spread knowledge that has been shaped by scientific communities. Access to this literature is essential for any scholar. Evidently,“biblio gifts” are easily accessible online without any charge from text-sharing platforms. Similarly, virtual groups in social networking websites such as Facebook, LinkedIn, Twitter and virtual blogs are used as pirated platforms. However little argument has been acknowledged regarding such platforms, especially concerning the size of the digital database, the major research areas covered and where these research articles come from (primary sources).

Complete possession of intellectual property for a large proportion of the scholarly database has been seized by a number of publishers. Many research articles are barricaded behind paywalls and remain unavailable for research scholars. In recent times, there has been an enormous expansion of open-access journals (OAJs). In 2009 alone, around 4,800 OAJs published approximately 190,000 research articles. However, despite expansion of this number to over 10,000 OAJs in 2015,^1^ Fuchs and Sandoval revealed that 88% of all the existing journals were still blocked and only 12% could truly be categorized as OAJs.^2^ Consequently, some readers have become encouraged to use pirate web platforms such as Sci-Hub and Library Genesis (LibGen), which enable free access to paid content such as journal databases and digital libraries, in order to access paid research content. Such malpractices can alternatively be regarded either as unethical and a criminal offense or as an act of civil disobedience.^3^

While piracy has eventually become an inevitable subject for debate within the domain of scholarly communications, the public experience of the Sci-Hub and LibGen platforms has stimulated the decade-old debate about the potential contribution of the commercial aspects of scholarly publishers, digital libraries and copyright towards creating an atmosphere within which the outcome of scholarly enquiry is uniformly accessible for all.

Sci-Hub and LibGen became known as infringing web platforms that allowed unauthorized pirated research content from copyright scholarly databases to be accessible. Their initial aim was to assist researchers who were unable to have institutional access to these digital libraries and were reluctant to pay the subscription fees per research article. These unauthorized web platforms, which can be considered to be shadow libraries, offer property and contravene access for academic purposes.^4^ High attention was drawn to them after a Kazakh computer programmer, Alexandra Elbakyan, who was recognized as the owner of Sci-Hub, faced enormous public criticism through a lawsuit filed by Elsevier against the unauthorized web platform and the programmer herself in a New York court (Elsevier Inc. et al. versus Sci-Hub et al. Case No. 1:15-cv-04282-RWS).^5^

To conclude, pirated web platforms like Sci-Hub and LibGen, along with some access support groups in social networking websites, e.g. Facebook, Twitter and all the virtual blogs, are considered by many traditional publishers to be a major threat. Many researchers find it irresistible to choose a simple interface in order to access a wide range of research articles. However, this is not a justification for using infringing web platforms, especially when morals within publication are a subject of deep concern from an ethical viewpoint.
